# A System of Dental Surgery

**Published:** 1860-05

**Authors:** 


ART. II.—*A System of Dental Surgery.* By JOHN TOMES, F.R.S., Dentist to the Dental Hospital of London, and to the Middlesex Hospital. With Two Hundred and Seven Illustrations. Philadelphia: Lindsay & Blakiston, 1859. 8vo., pp. 668.

*A Practical Treatise on Operative Dentistry.* By J. TAFT, Professor of Operative Dentistry in the Ohio College of Dental Surgery. With Eighty Illustrations. Philadelphia: Lindsay & Blakiston, 1859. 8vo., pp. 378.

CONSCIOUS of the great void in dental literature, and especially in the department proper, of which the first work professes to teach, we were pleased to hear of its forthcoming. The character that Mr. Tomes had established for himself in the publication of his "Course of Lectures on Dental Physiology and Surgery," delivered at the Middlesex Hospital School of Medicine, was a sufficient guarantee to us of the value of the expected addition to our literature.

For a quarter of a century the author has been stimulated in his investigations by the equally intelligent and persevering researches of an Owen and a Nasmyth, and the valuable contributions which he and his colaborers have made to science have long since been acknowledged. Appreciating as fully as any one the value of the minute study of all things pertaining to a science such as that of dentistry, we fear that the tyro and those for whom text-books are required, will not reap the same advantage from this treatise that they would have done had the author made it less microscopic in its character and endeavored to impart to it more of what it purports to be—a system of dental surgery. It is true, the entire science of dentistry is denominated the science of dental surgery, even that part which is evidently mechanical, and which treats of filling teeth; but we think the time has arrived when an entire separation of the different branches is required, so that they may be more fully and ably taught. It is so in the science of medicine, and with equal propriety ought to be in dentistry.

In the work under consideration two hundred and eighty pages are devoted to an examination of the different periods and conditions of teething, and while there are in these pages comparatively few points wherein we are disposed to differ from the author, we regret to see so much of a book, containing in the whole only six hundred and sixty-eight pages, taken up by matter so far foreign to the subject. Admitting the necessity of a proper understanding of everything that pertains to a science, by those who fain would enter its portals, we heartily commend the zeal and industry of those who, aided by all the inventions that art has produced, have "grown pale by the midnight lamp," in their desire to expose to light, in their day and generation, those things which so long have been hidden from man. And while we are disposed to hoard up and keep in their proper places all such revelations, we desire to see the day when the science of dentistry shall have a reliable literature, wherein each department shall be separately treated and plainly taught.

There is a small portion of matter included under the head of "teething" that we are disposed to screen from the charge of intrusion, as the subjects treated properly belong to a system of dental surgery; we mean that part which relates to the *abnormal* conditions of the teeth and the dental arch, and the irregular eruption of the teeth; and of this we are happy to say that the author has fully demonstrated the fact that serious disease, as well as great deformity, is often induced by the irregularity of the teeth, both before and after their entire eruption.

The portion of the work devoted to "the dental tissues" is very good, and a perfect understanding of it very essential to a correct treatment of these tissues when diseased; but as the dental tissues can only be considered in a pathological point of view, in a work of this character we would expunge, or, rather, place in their proper departments, these together with all other matters that take a different direction from the subject under consideration.

The article on "caries" is valuable, logical, and scientific, and recommends itself to the careful study of all who wish to make themselves acquainted with a subject that has been "a bone of contention" for years. The rules laid down for the treatment of caries are such as no one can object to, and it were well were they properly understood by all. The instruments recommended for, as well as the materials used in, filling, are such as are found in the hands of the well-informed dentists of our own country.

To the remainder of the work we could cheerfully annex our sign-manual, as we feel that its efforts are in the proper direction; and while we heartily commend the entire work to the kind consideration of the profession, conscious that its influence will be greatly felt, we must confess that we sincerely regret that Mr. Tomes did not furnish to us that which we so much need, a *complete* system of dental surgery. The distinguished author, it is but justice to add, has supplied us with a model of composition at once pure, chaste, and classical; and in taking leave of him, we beg to declare that he has supplied us with one of the most readable scientific books in the English language. The engravings are nearly all admirable, and greatly enhance the value of the work.

The work of Professor Taft, unlike the one above noticed, is strictly confined to the subject which it professes to teach; and, were it not for certain defects, which we regret to say are both of a scientific and literary character, we should hail its advent as one calculated to add much to our literature, and as initiatory of a system much needed.

As this is an American publication, we notice these defects with regret, and our mortification is increased by a knowledge of the fact that it is not through ignorance of the author that these objectionable points present themselves, but rather because of a want of caution and reconsideration.

The author's character as an operator, and as one anxious for the advancement of his profession, is second to none, and being fully competent to the task he undertook, we would much prefer to be able to give to this volume such a welcome as would be gratifying to all concerned; but an honest sense of duty to science bids us lay aside all personal feelings, and to regard not individuals, but treat that which is presented for the consideration of a liberal profession upon its own intrinsic merit.

A desire to prepare manuscripts for publication within a given time, in compliance with the wishes of publishers, should never induce an author to be precipitate, and thereby place in jeopardy his reputation. We do not pretend to say that this was the case in this instance, but certain it is that, by some cause or other, the work appeared prematurely, and while a little more care might have made it all that could be desired, it can now only be considered as a primary work for students, containing much that will be of value, together with some things that might lead them into error.

Lest we should be deemed unjust in our remarks, and charged with making assertions without foundation, we will cite a few instances wherein we consider it defective.

At page 55 the author states that "the teeth cannot, with impunity, undergo sudden transitions from one extreme of temperature to another, *or even such extremes as may be endured by the surrounding parts.* By these *inflammation of the dentine* may be induced and the vitality of the teeth diminished, *so that even in friable teeth* checking of the enamel will occur, and thus a condition arise that will facilitate decay."

We unhesitatingly declare that this position cannot be sustained. Teeth have been subjected to the most severe tests, as far as the transition from one extreme of temperature to another is concerned, and nothing has been elicited that would at all substantiate the above theory. Again, such a condition as "*inflammation of the dentine*" has never been satisfactorily demonstrated; nay, the possibility of its existence is even boldly denied by very good authority.

Another point in this paragraph, and we have done with it: "so that even in *friable* teeth checking of the enamel will occur." Will not the student ask why the author says "*even in friable teeth,*" etc., when he could have established his doctrine much better by proving that even the most *resisting* teeth are broken?

At page 72 we find the orthodox doctrine set forth, that "the filling of the teeth is predicated upon the nature of decay, upon the fact that the lost portion will not be restored by nature, and upon the fact that caries is an effect of external causes, and not of any cause within the tooth itself." This is all very well; but if external causes alone produce decay, why speak of inflammation of the dentine, as in the above connection?

Two of the most glaring oversights of the author, to which our atten- has been directed, occur, the one at the bottom of page 89, the other on page 218. In the first he is made to say, whether through the treachery of printer's ink or otherwise, we are unable to determine, that "amalgam fillings in a short time after their insertion undergo a hardening process, *caused mainly by evaporation of the mercury.* The consequence is, either that the mass becomes porous, or that it contracts; the former, doubtless, in cases where the oxydation blackens through, and the latter, where it is confined to the surface."

That amalgam fillings harden, we do not deny, but that this process is brought about by the "evaporation of the mercury" we do most unhesitatingly dispute. When two or more metals unite, forming an amalgam, they no longer preserve their distinguishing characteristics, but form a new compound, which is solidified by *crystallization*, as is demonstrated by the heat evolved during solidification and the crystalline appearance when fractured. As the amalgam is rendered hard very soon after its insertion the *evaporation of the mercury*, did it even occur, could not cause the filling to contract; and if it is by this means that the filling is rendered porous, would it not crumble at once? On the other page referred to, the author would appear to consider *arsenious acid* as a substance possessing *vitality*. In speaking of its escharotic properties, after referring to the views of Professor Bache and Dr. Pereira on this subject, he says that "*its escharotic power certainly bears no proportion to its vitality;* but it is probable that it forms definite compounds with some of the constituents of living tissue; and if so, these compounds appear to be readily and rapidly decomposed, so that the acid becomes again *free* to attack, with similar results, the subjacent parts."

While the first proposition is certainly not in accordance with the teachings of chemistry, which recognizes arsenious acid as an oxyd of an elementary substance, inorganic, and *non-vitalized*, the last may be considered as one of its nice theories, which it would be more difficult to demonstrate than to force upon the minds even of the credulous.

Chapter fourth, devoted to instruments for filling, is finely illustrated with valuable representations, and is calculated to give to the student, and even to the practitioner whose views are antiquated, a correct idea of such instruments as will be almost indispensable to him. Directions are also given as to the proper mode of manufacturing instruments; this is important, as no author is able to present all the forms and varieties that are in use, or that the peculiar taste or mode of operating of each individual may require.

We trust that what we have said in regard to the above works will not be attributed to a spirit of fault-finding. In reference to the last noticed, we are fully aware that there are few inaccuracies that would not have been discovered by its author had he examined it a little more carefully, and while we look upon book-reviews as calculated to stimulate future authors to greater watchfulness, rather than to condemn works already written, we trust that none may be deterred from contributing their part toward establishing a reliable literature, and that all will feel the responsibility of the position they occupy in the profession.

It is not possible for any writer to produce a work that may not be objected to by some one; and while our constant aim should be to present our ideas in a form that would be least objectionable, the fact that the products of our labors may be roughly handled by the critics should not prevent us from making the effort.

The mechanical execution of both of these books is unexceptionable, and reflects great credit upon the Philadelphia publishers.

As the science of dentistry has at last reached that culminating point from which it is able to maintain its position among the learned professions, we shall append a short history of its progress in the United States.

Although our country cannot boast of having given birth to the science, her sons at least have the credit of having brought it to its present state of perfection; and could our laws afford a little more protection against the inroads of quacks, the time would not be far distant when the escutcheon of dentistry would be as unblemished as that of any known science. It is only in reputation that the profession is injured by these arrant impostors who infest the country; for while their bad deeds bring into discredit that which time has proven to be a *true* science, these same cases of malpractice require competent hands to supply a remedy. The efforts now made by those who have a sincere love for their profession, although unaided by legal patronage, and prompted by the purest motives, are slowly but surely bringing about the anxiously expected period when the grim visage of the vile pretender—sometimes concealed behind the bland countenance of the apparent gentleman—shall show forth so plainly that none may be deceived: a change effected not by oppressive persecution, but by an onward, upright discharge of duty.

That the dental art has made more rapid advances in this country than elsewhere, is admitted by all; even our ever-ambitious and scientific brethren in England give us the credit of pushing onward and upward, with energetic hands, the standard of the dental profession. They attribute our success in some measure to the native energy of character for which Brother Jonathan is so renowned, but at the same time acknowledge the influence of the abolition of those small jealousies and invidious distinctions with reference to less fortunate brother practitioners, which allows a free communication of ideas among the members of the profession, and which has been operative in the production of associations whose objects are to cultivate the science of dentistry and all its collateral branches, to elevate and sustain the professional character of dentists, and to promote among them mutual improvement, social intercourse, and good feeling.

Although the profession here has been diligent in this particular, there has been as yet very little done toward the production of a literature such as the rapid march of dentistry would indicate, and such as is absolutely required.

Before referring to the long list of books that have been brought before the public, some of them with no other evident object than to introduce their authors into notice, we shall take a glance at the early age of dental surgery; and, if time and space would permit, we think we could show that, notwithstanding its present extended and rapidly enlarging proportions, it has not "grown too quickly great to grow well."

As early as the time of Hippocrates, we meet with distinct notices of the diseases of the teeth. In the sarcophagi of the Egyptians rudely manufactured teeth have been discovered; and proof of the fact that gold had been made use of as a stopping for cavities, has been obtained by the examination of some mummies from Thebes. Historical evidence is also abundant that the general appearance of the teeth and their diseases attracted considerable attention among the Greeks and Romans.

The first work specially devoted to the dental art was written by Guantier Henry Ryff, in the year 1548. From the writings of Martial, Catullus, Lucian, and Pliny, we may infer that that branch of the science designated as mechanical dentistry was not unknown to the ancients; for, although no mention is made of the replacement of the natural teeth by artificial ones by the Greek and Roman scientific writers, there is no doubt that at the period they wrote, and for a considerable time previously, this branch was extensively practiced at Athens and at Rome.

The following quotations from Martial leave no doubt that the Romans used artificial teeth :—

> "Sic dentata sibi videtur Œgle
> Emptis ossibus, Indicoque cornu."—*Martial*, lib. i. 72.

> "Thais habet nigros, nivios Lecania dentes,
> Quæ ratio est ? emptos hæc, habet, illa suos."—*Martial*, lib. v. 44.

As a notice of the progress of the science of dentistry in our own country will be of more interest to our readers than a review of its struggles in the darker ages, we shall take up for consideration, in the first place, that part of the profession which, while it enables the operator to display his handiwork, and is of marked benefit to those who have been deprived of their teeth, is nevertheless regarded by many of minor importance to that branch which rescues from destruction organs which man, with all the aid that science can afford, can never replace.

We do not wish to be understood as intending to cast odium upon the mechanical department of the science,—for we are of the opinion that it requires as thorough an education of the head as well as the hands to make a good *mechanical dentist,* as to qualify one to perform operations upon the living teeth; but, while we admire the exquisite degree of perfection to which this branch has been brought, we fear that too great reliance is placed upon the powers of man to imitate the Creator, and many valuable teeth are sacrificed, or allowed to go to destruction, that might have been rendered useful by the skillful manipulations of a surgeon-dentist. And while we admit that clear-sighted, energetic, mechanical dentists have rather outstripped those in the other branch in the race of improvement, we can not lose sight of the fact that within the present century dental surgery has become so nearly a perfect science that those who have been fully initiated can confidently promise entire success for all their operations. The microscope has thrown so much light upon the study of the pathological and physiological conditions of the teeth, and the improvements in material for filling, and in instruments for the proper performance of all the operations upon these organs have been so great, that it would appear that little remains to be done in this direction.

It is said that the first set of artificial teeth ever worn in this country was made in Paris, and was in possession of Aaron Burr.

The manufacture of porcelain teeth is modern in its origin. Originally natural or human teeth were used; also calves' and sheep teeth; next ivory, or teeth carved from the tusk of the hippopotamus. Fifty years ago there was not a porcelain tooth made in this country. Twenty years ago not more than two hundred and fifty thousand were manufactured annually in the United States, and but a trifling number in Europe. Since then the demand has been continually increasing, owing in a great measure to the rapid improvements made from year to year, and the more perfect applicability to the purposes designed; and within the last eight years, it is said the consumption has increased one hundred per cent.

Philadelphia was the first and original seat of the manufacture of porcelain teeth in the United States; and over one-half, if not two-thirds of all the teeth now made in this country, are made in Philadelphia. One firm, that of Jones, White & McCurdy, 528 Arch Street, make annually about 1,250,000 porcelain teeth. Their products are exported to Europe, South America, the West Indies; and in all the markets they take precedence over the European manufacture. * * * * * The statistics of the manufacture of porcelain teeth, as furnished to us, are as follows :—

| | |
|---|---|
| Number of establishments - - - - - - | 5 |
| Capital invested - - - - - - - - - | $175,000 |
| Persons employed, one-half females - - - - | 125 |
| Value of production annually* - - - - - - | $500,000 |

The facts in relation to the superiority of the porcelain teeth of American manufacture still hold good, and the establishment in this city, which has won for itself such a wide-spread reputation, through the skill, enterprise, public spirit, and gentlemanly deportment of the proprietors, still exists.

The number of establishments for the manufacture of teeth is the same as when the above was written, but the capital invested at this time is not less than $300,000. The number of persons employed is about 175, and the value of the annual production about $750,000.

Although mineral teeth were introduced into this country early in the present century, and some experiments made in their manufacture about the year 1825 or 1826, all who are at all acquainted with the profession prior to the year 1831 will remember that the teeth made, up to that time, were of an opake clay body, and more after the appearance of split beans, both in color and shape, than anything else they could be compared to; this was especially true of the French teeth.

The experiments made at that time, in this city, were conducted by Messrs. Joseph E. McIlhenny, Charles Peal, the artist, then in his eightieth year, and Mr. Plantou the elder. Dr. Hudson, who then had the most extensive practice in Philadelphia, depended upon teeth of French manufacture, which, although very rough, were rather better than those made in this country.

The difficulties encountered by the above-named experimenters were sufficient to cause many of the present day to abandon as hopeless any similar undertaking; being entirely unaided in their efforts, both as regards modes of manipulation and localities from whence to procure their materials, their progress was necessarily slow; and, when viewing the results of their labors with a second sober thought, it ought not be a matter of so great gratulation that operations can now be performed with so much perfection, knowing that we reap that which was sown in vexation and disappointment by those who preceded us.

Notwithstanding the French were the first to introduce porcelain teeth to the profession, the sequel has shown that they have not kept up with the times in improvement, both in that direction and also in the surgical and operative departments; for we of "the land of sober thoughts and quick invention," have, by taking advantage of their masterly inactivity,

* Philadelphia and its Manufactures, pp. 398 and 399; 1858.

been able to carry our reputation into their very capital, and from thence entirely over Europe.

The superiority of the operations of American dentists over all others has been acknowledged by the crowned heads of the Old World, and the fact that students cross the Atlantic, yearly, in order to receive instructions at our colleges, is ample evidence that we possess *character* as well as reputation.

It were useless to consider in detail the numerous modes of constructing artificial dentures, and to examine the many and complete contrivances for the correction of irregularities; suffice it to say, that, notwithstanding the yearly additions that are made in the way of invention, the general principles that govern the actions of the educated remain the same, and it is because of these fixed principles that improvements are made.

In the operative department, the laws of physiology and pathology govern all those who operate for the patient's good, as well as their own emolument.

The careful study of the minute structure of the teeth is so strongly impressed upon the student, that all who enter the profession through legitimate channels are able to proceed understandingly.

The material now used for filling teeth is principally gold, and the article of gold-foil manufactured in this country equals, if not surpasses, that of foreign make. The amount of gold consumed in this way, and in the form of plates as bases upon which to fix artificial teeth, is almost beyond conception. One firm alone, in this city, that of Charles Abbey & Sons, 228 and 230 Pear Street, the senior partner of which is the original manufacturer of gold-foil for dentists' use, in this country, consumes an immense amount. These gentlemen refine all of their gold themselves, and we are credibly informed that they melt from $5000 to $8000 at a time.

It was through the persuasion of the distinguished Dr. Hudson that Mr. Abbey was first induced, about the year 1817 or 1818, to manufacture dentists' foil; prior to that time he was engaged in the manufacture of gold-leaf for gilders' purposes. The principal dentists then in this city were Hudson, Harrington, Gardett, and Gilliams; these have all left for themselves imperishable names as operators, although they labored under countless difficulties.

About the year 1829 or 1830, so great was the increased demand for gold-foil that Mr. Abbey abandoned the manufacture of gold-leaf, and from that time to this the foil of his make has been in great request. The reputation that he acquired in the days of Hudson and his compeers has not been in the least diminished, nor is it likely soon to be; for we can confidently say of the present firm, that, combined with undoubted skill as manufacturers, they possess that which is of equal value to those engaged in a business such as theirs, an umblemished reputation for integrity.

From reliable statistics we are enabled to compute the amount of gold consumed by the dental profession in this country at about *two and a half millions of dollars* annually. This, we think, is an under-estimate, and is allowing for all that would be likely to come back to be reworked.

We agree with the editor of a late number of a dental journal, that there is some ground for the idea "that the mines of gold, a century hence, will be sought for in the cemeteries of the old cities, where it has been geologically deposited under the industry of dentists."

The number of dentists now practicing in the United States, as nearly as we can ascertain, is about five thousand, which would give—computing our population at only twenty-five millions—one dentist to every five thousand inhabitants; and as many of these belong to the unfortunate class who possess "skulls that cannot teach and will not learn," it will be seen that there is ample room in the profession for all those who will fully equip themselves for the responsible position, and foster the commendable ambition to be foremost in the ranks.

This fact should encourage many young medical men, who have waited in vain for employment, to acquaint themselves with the science, and while they are acquiring an honorable competency, aid in driving from a rapidly advancing profession those who are unworthy of its honors and benefits. Men possessing a sound medical education are wanted in the dental ranks; without a fair acquaintance with anatomy, physiology, materia medica, chemistry, surgery, and therapeutics, *no man can be an accomplished dentist;* he cannot meet all the exigencies of his position, and perform with safety its highest duties, much less can he advance the science toward that excellence which it still demands from its disciples. It is true, we now have in this country three flourishing colleges, wherein a thorough dental education is imparted; but none are so ripe for the highest honors as those who have first had a careful medical training. Among the schools which now exist, the Baltimore College holds the rank of pioneer, the Ohio College comes next, and the one which is located in this city—the centre of medical learning in this country—although last in entering the lists, is not least in its efforts to merit the confidence of the public, by sending out only those who are qualified and true to their calling.

Among the alumni of these institutions may be found those who have arrived at distinction in the profession, both by the superiority of their operations and their valuable contributions to the journals,—of which, we are proud to say, we have a number that are in a measure supplying the great want, as far as the right kind of books is concerned.

The colleges, although not supported as they should be, are steadily growing into notice and reputation; those who have gone forth from their walls have demonstrated so fully the benefits they have derived, that many who have long been connected with the profession in an illegitimate way are now seeking admittance through the schools, as the most direct channels to successful business. Some who are at first attracted by the honors of the doctorate, enter in order to obtain that, but they are very soon made sensible of the fact that there is more to be obtained than a title, and by attention to the instructions imparted they become all that could be desired.

Of the periodicals to which we have referred, the *American Journal of the Dental Sciences*, which made its appearance in June, 1839, and was originally conducted by E. Parmly, E. Baker, and Solyman Brown, as publishing committee, and during the first year of its progress under the editorial charge of E. Parmly, of New York, and C. A. Harris, of Baltimore, in which city it was printed, is the oldest dental journal now issued. During the first year of its existence it was issued with some irregularity, in monthly numbers, at three dollars annually. At the close of the year it became the property of the American Society of Dental Surgeons, then just organized. The title was changed to that of the *American Journal and Library of Dental Sciences;* it was issued quarterly instead of monthly, and the subscription price was increased to five dollars. The Society placed it in charge of C. A. Harris, of Baltimore, and Solyman Brown, of New York, who were to be assisted by a regular corps of contributors. From that period to August, 1850, the journal continued to be issued under the auspices of the Society, under the charge of several editors, appointed annually by the Association. At the meeting of the Society in 1850, it was transferred to Professor C. A. Harris, of Baltimore, and it is now a private enterprise.

That this journal has done good service to the profession, all are free to admit; and we think that, together with two or three other periodicals of more recent date, it has done more to disseminate sound knowledge among the members of the profession than nearly all the "Popular Treatises" on dental subjects that have made their appearance, many of them unfortunately merely as advertising mediums for their authors. Two other journals which appeared about the same time, in the year 1847, the *Dental Register of the West*, issued quarterly, and published under the auspices of the "Mississippi Valley Association of Dental Surgeons," and the *Dental News Letter*, published quarterly by Jones, White & McCurdy, Philadelphia, have contributed greatly to the diffusion of information.

The former still exists, but has become the property of J. T. Toland, Cincinnati, Ohio, and is ably edited by J. Taft and George Watt; it is also changed from a quarterly to a monthly; and by the racy style of its editorials, and the valuable information diffused through its pages, it is rapidly gaining in favor. We would suggest, however, to the editors of this journal,—if our advice is of any value,—the total exclusion of undignified articles, similar to that under the caption of "D. M. C.'s Reply," etc., in a late number. We are not disposed to tie down journals, although they profess to be scientific in their character, to the staid forms represented by those conducted by theological professors, but we do think that *morality* alone should demand the suppression of articles such as the one above referred to.

The *Dental News Letter* continued its existence up to August, 1859, and during all that time was ably conducted by Drs. White and McCurdy; with the exception of the occasional admission into its pages of personal and unprofessional articles, its entire career was one of marked success. We are happy to state that during the latter part of its existence the objections above referred to did not occur. On the 1st of August, 1859, the *Dental Cosmos,* a monthly journal, published by Jones & White, Philadelphia, and edited by J. D. White, D.D.S., J. H. McQuillen, D.D.S., and Geo. J. Ziegler, M.D., took the place of the *Dental News Letter.* The known ability of these gentlemen as writers, and the prominent positions they occupy in their respective professions, are sufficient guarantees of the character of the journal in their charge; ever zealous for the advancement of the profession, we feel satisfied that they will leave nothing undone to promote its interests.

The elevated tone that has characterized this "monthly," thus far in its career, is strongly indicative of a healthy state of the science; and, profiting by the experience of the "quarterly," which it has displaced, we doubt not the profession may rely upon this as a medium through which to obtain that kind of intellectual nourishment which will build it up.

The periodical literature of a science possesses advantages over works of any other character, whether monographs or compendiums, from the fact that it is more widely circulated; and the individual articles being comparatively short, are read and studied with greater ease than the more elaborate productions. While text-books are greatly wanted, as well as works entering into the minutiæ of every subject connected with a science, that portion of the literature which visits the student or operator at times when he is not disposed to study, and is yet loth to be idle, imparts information that is not soon forgotten. The fact that journals fill the place—sometimes for a considerable period—of an extended library, in the offices of the poorer members of the profession, should remind those who send them forth of the importance of furnishing nothing but that which is of value. Knowing that good *can* "come out of Nazareth," let them hope that some naturally bright mind may be reached even in the darkest corner of the earth, whose contributions to science, in after-life, will compensate in full for all the labor they may bestow in protecting their pages from the detracting effects of unprofessional articles.

We had intended to refer in detail to the other works that have been written from time to time, and brought before the profession, but as very few of these, with the exception of the works of Professor C. A. Harris, of Baltimore, have met with even a moderate degree of favor, we will not weary our readers by directing their attention to them, but close our observations upon the dental profession and dental literature with the hope that no very great time will elapse before we shall be called upon to notice some marked evidence of the continued advance of a science the prosperity and ultimate triumph of which we shall hail with the highest gratification.

